# Antiangiogenic potential of *Elaeagnus umbellata* extracts and molecular docking study by targeting VEGFR-2 pathway

**DOI:** 10.1515/med-2024-1083

**Published:** 2025-01-16

**Authors:** Nausheen Nazir, Arbaz Waqar, Amir Zaib Khan, Ayaz Ali Khan, Tariq Aziz, Abdullah F. Alasmari

**Affiliations:** Department of Biochemistry, University of Malakand, Chakdara Dir Lower 18800, Pakistan; Department of Biotechnology, University of Malakand, Chakdara Dir Lower 18800, Pakistan; Laboratory of Animal Health Food Hygiene and Quality, University of Ioannina, Arta, Greece; Institute of Molecular Biology and Biotechnology, The University of Lahore, Punjab, Pakistan; Department of Pharmacology and Toxicology, College of Pharmacy, King Saud University, Riyadh, Saudi Arabia

**Keywords:** *Elaeagnus umbellata*, antiangiogenic effect, CAM assay, molecular docking

## Abstract

**Background:**

Anti-angiogenesis or inhibition of blood vessel formation is the best way to prevent the growth and metastasis of tumors. Natural sources like plants are currently being explored for its antiangiogenic activity as they are factories of various phytochemicals. The goal of the current study is to investigate the antiangiogenic potential of *Elaeagnus umbellata* (*E. umbellata*) by using chorioallantoic membrane (CAM) assay and molecular docking.

**Methods:**

Based on our previous research, the antiangiogenic activity was carried out using active fractions including crude methanol (Eu-Met), ethyl acetate (Eu-EtAc), and chloroform (Eu-Chf) extracts using CAM assay. Furthermore, to understand the binding mechanism of identified compounds, molecular docking was performed against vascular endothelial growth factor receptor 2 (VEGFR-2) using AutoDock vina as docking software. VEGFR-2 is overexpressed in pathological angiogenesis.

**Results:**

In CAM assay, Eu-Met, Eu-EtAc, and Eu-Chf extracts showed antiangiogenic activities but notable antiangiogenic activities were exhibited by Eu-Chf with IC_50_ value of 65.02 μg/mL. In molecular docking, five compounds, catechin, catechin hydrate, morin, quercetin, and rutin, reported in the extract and active fractions (Eu-Met, Eu-EtAc, and Eu-Chf) of *E. umbellata* showed strong interactions with VEGFR-2 with binding affinities of −9.4, −9.3, −9.9, −10.2, and −9.4 kcal/mol.

**Conclusion:**

Based on our results, we can claim that *E. umbellata* possess antiangiogenic activity which needs to be explored further.

## 1 Introduction

Angiogenesis, an important aspect of human physiology, is a process in which new blood vessels arises from pre-existing blood vessels which help in wound healing, growth, and embryonic and organ development [1]. However excessive angiogenesis can also occur in a variety of conditions like cancers, and cardiovascular diseases [2]. In the tumor microenvironment rapid cancerous cell growth creates an urgent need for a constant blood flow and nutrients. Angiogenesis provides all the necessary nutrients for cancer/tumor cells growth [3,4]. Judah Folkman postulated that cancer/tumor cells strop spreading when the blood vessels present in it are prevented or blocked [5]. Blocking blood vessels may prevent tumor growth. The process of angiogenesis is controlled by a complex network of growth factors and receptors. The vascular endothelial growth factor (VEGF) family of proteins, VEGF-A, VEGF-B, VEGF-C, VEGF-D, and VEGF-E, and its receptors, vascular endothelial growth factor receptor 1 (VEGFR-1), VEGFR-2, and VEGFR-3, play important roles in both pathological and physiological angiogenesis [6,7]. VEGF-A/VEGFR-2 signaling pathway has been recognized as the most critical factor in promoting angiogenesis and its interaction leads to endothelial cell proliferation, migration, survival, and new blood vessel formation by the activation of phosphorylation cascade that triggers downstream cellular signaling pathways, including the phosphatidylinositol-3 kinase/protein kinase B (PI3K/AKT) and rapidly accelerated fibrosarcoma/mitogen-activated protein kinases (RAF/MAPK) pathways [4,8,9]. Certain cancer like endothelial cells of neovascular tumor, malignant melanoma, B-cell lymphoma, lung, urothelial, breast, colorectal, and other cancer cells have all been found to overexpress VEGFR-2 [10,11]. Therefore, VEGFR-2 is an attractive target for target therapy. At present, numerous VEGFR-2 inhibitors have been created or are in various stages of development; sorafenib, cabozantinib, and sunitinib are widely used in clinical cancer treatment, But still, new inhibitors are needed [12].

Medicinal plants are widely used to treat various disorders [13]. They are regarded as rich resources of traditional medicines and from these plants, many of the modern medicines are produced [14]. Medicinal plants are valued much as they are factories of natural products and they produce a variety of phytochemicals such as carotenoids, phenolic acids, phenols, and flavonoids that have exhibited effective biological potentials [13]. Several phytochemicals have been effectively tested against cancers after being derived from various plant species [15,16,17,18]. *Elaeagnus umbellata* Thunb. (*E. umbellata*) is a berry fruit plant which has high medicinal values. It belongs to the Elaeagnaceae family and it is native to Central Asia and southern Europe [13]. Historically, the Elaeagnus species have been used as antimicrobial, antidiabetic, antimutagenic, antioxidant, anticancer, antiulcerogenic, anti-inflammatory, neuroprotective, and antinociceptive agents [19,20,21]. The *E. umbellata* fruit/berry are rich in vitamins A, C, and E, minerals, flavonoids, alkaloids, steroids, terpenoids, saponins, essential fatty acids, etc., and phenolic acids (cinnamic acid and benzoic acid) and flavonoids (epigallocatechin gallate, myricetin) [20]. Furthermore, *Elaeagnus* fruits/barriers also contain some bioactive compounds like lutein, phytofluene, phytoene, β-carotene, β-cryptoxanthin, and α-cryptoxanthin [20]. Various extracts and isolated compounds like catechin, chlorogenic acid, epigallocatechin, epigallocatechin gallate, ellagic acid, morin, pyrogallol, quercetin, and rutin of *E. umbellata* have previously been investigated for their anti-diabetic, anticholinesterase, and antioxidant potentials [22]. Research studies reveal that no prior research has been done on the antiangiogenic activity of *E. umbellata*. In this study, we investigated the antiangiogenic activity of *E. umbellata* by Chorioallantoic membrane (CAM) assay and we also investigated the interactions of 12 compounds that were identified in the *E. umbellata* crude methanolic (Eu-Met) extract and fractions (ethyl acetate [Eu- EtAc] and chloroform [Eu- Chf]) against human VEGFR-2 by using a molecular docking approach.

## 2 Materials and methods

### 2.1 Plant collection and extraction/fractionation

The study focuses on the extraction/fractionation of the medicinal plant *E. umbellata*. The plant was collected in August and September, 2023 from Kalam, District Swat, Khyber Pakhtunkhwa, Pakistan, and authenticated by botanical taxonomist Dr. Gul Rahim and the plant specimen was deposited in the Herbarium of the Department of Botany University of Malakand. After being crushed, the dry fruits were macerated and filtered. A solidified crude Eu-Met was produced by utilizing a rotary evaporator to concentrate the filtrate. Solvent extraction was then used to fractionate the crude Eu-Met. After being suspended in distilled water, the crude Eu-Met was divided into various solvents. Every fraction (Eu- EtAc and Eu- Chf) evaporated to form masses that were semisolid [20].

### 2.2 Chick CAM assay

The CAM assay was used to detect the inhibitory effects of the test samples (extract/fractions) on the development of blood vessels [23]. Domestic chicken eggs were bought from a nearby poultry seller near the University of Malakand and fertilized by incubating them at 37°C for 4–6 days in a humidified incubator. Using a flash light, the development of blood vessels was verified after the incubation period. The egg’s narrow end was punctured, and a sterile syringe was used to remove approximately 1 µL of albumin. The yolk sacs were then separated from the shell membrane. On the eighth day of the experiment, a thermanox cover slip preloaded with the requisite concentrations of test samples (extract/fractions), and control drug was placed on the CAM surface and incubated for an additional 3 days. Later, a 33-gauge needle was used to inject acetone and methanol (1:1) into CAM, alienating it from eggs. The number of blood vessels in CAM was seen and quantified in all groups using a microscope. Six eggs were utilized for each test sample in this investigation. Normal saline was used as negative control while dexamethasone was used as positive control. The % inhibition of angiogenesis test was performed using the following formula:

(1) \[ \% \hspace{0.25em}{\mathrm{inhibition}}=\frac{{{\mathrm{CAM}}}_{{\mathrm{ns}}}-{{\mathrm{CAM}}}_{{\mathrm{ts}}}}{{{\mathrm{CAM}}}_{{\mathrm{ns}}}}\times 100,]\]

where CAM_ns_ is the number of blood vessels treated with normal saline and CAM_ts_ is the number of blood vessels treated with test samples.

### 2.3 Molecular docking

VEGFR-2 kinase domain 3D structure (3VHE) was retrieved from the Protein databank (PDB) [24,25,26,27,28]. It was further purified, i.e., water and its ligands were removed by using Discovery Studio 2021 (Accelrys Software Inc., San Diego, CA) [29] and it was saved in PDB format. The prepared protein was further opened in AutoDockTools-1.5.7 [30], Polar hydrogens, Kollman charges, Gasteiger charges, and AD4 type atoms were added to it and the protein was saved as a macromolecule in Protein Data Bank, Partial Charge (Q), & Atom Type (T) (PDBQT) format. The grid box then defined the protein’s (3VHE) active site with the following dimensions −25.517, −0.517, −9.228, and a configuration file was prepared. The ligands/identified compounds used in this study were selected from the reported study [20]. Their 3D structures were downloaded from PubChem database [31]. The ligands were also then opened in AutoDock-1.5.7, their roots were detected and their number of torsions selected and were saved in PDBQT format. For docking simulation AutoDock vina was used [32,33].

## 3 Results

### 3.1 Chick CAM activity

In CAM assay, notable antiangiogenic activities were shown by Eu-Met extract and fractions (Eu-EtAc and Eu-Chf) of *E. umbellata,* which are shown in Table 1.

**Table 1:** Antiangiogenic potential of Eu-Met extract and fractions (Eu-EtAc and Eu-Chf) of *E. umbellata*

Samples	1,000 μg/mL	500 μg/mL	250 μg/mL	125 μg/mL	62.5 μg/mL	IC_50_ μg/mL
Eu-Met	70.22 ± 0.42	63.77 ± 0.28	56.58 ± 0.93	43.87 ± 0.46	38.62 ± 0.47	**191.64**
Eu-EtAc	73.77 ± 0.28	66.92 ± 0.35	54.67 ± 0.20	46.25 ± 0.24	42.03 ± 1.04	**157.55**
Eu-Chf	78.05 ± 0.48	70.30 ± 0.89	64.25 ± 0.24	55.95 ± 0.24	51.00 ± 0.58	**65.02**
Dexamethasone	89.92 ± 0.35	82.03 ± 1.04	75.05 ± 0.48	67.77 ± 0.28	61.70 ± 0.30	**29.74**

Comparisons were made between standard (Dexamethasone) and extract (Eu-Met, Eu-EtAc, and Eu-Chf) treated groups using one way ANOVA followed by Dunnett s posthoc multiple comparison test (**p < 0.01, ***p < 0.001).

**Figure 1:**
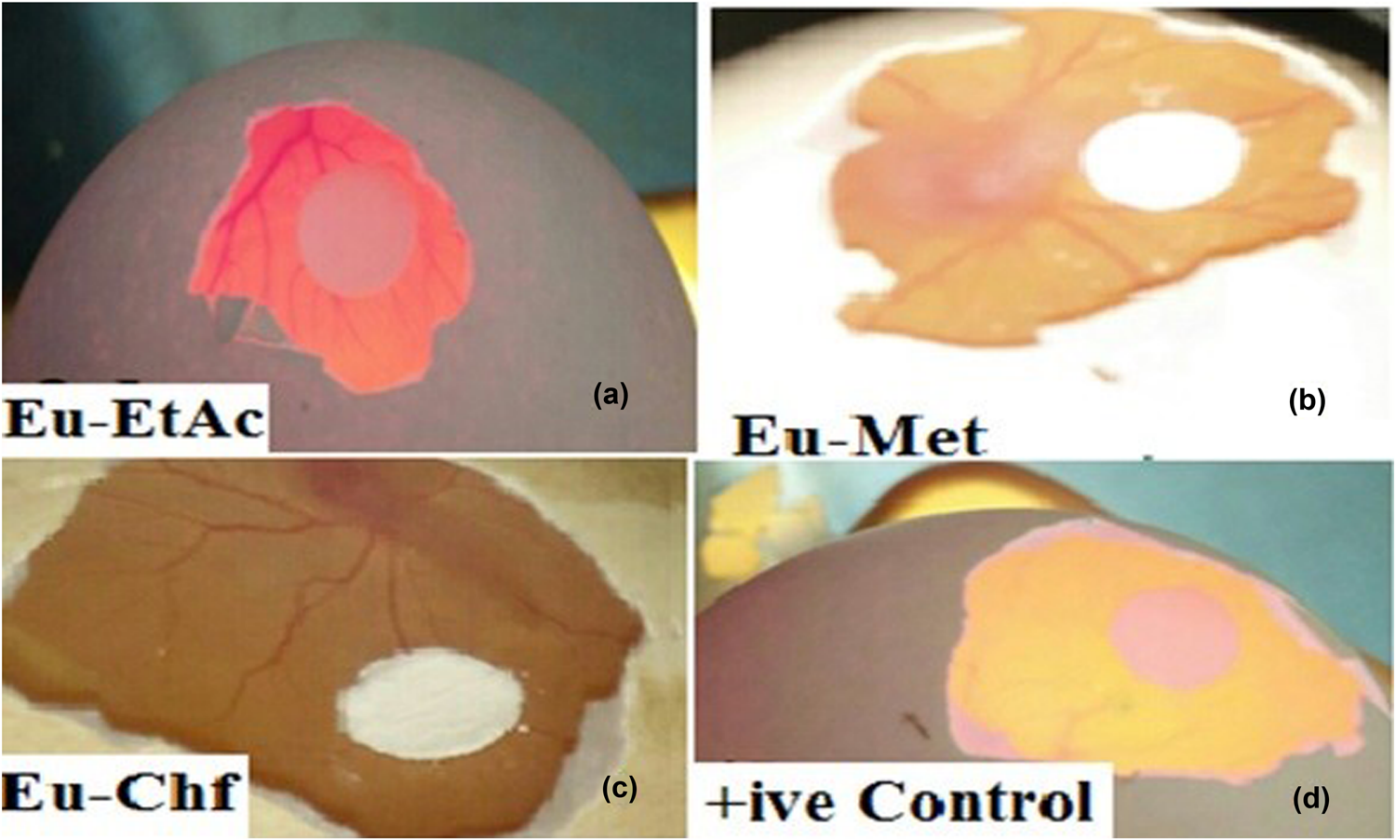
Antiangiogenic potential of *E. umbellata* in CAM assay. (a) Eu-EtAc, (b) Eu-Met, (c) Eu-Chf, and (d) positive control (dexamethasone).

Eu-Chf exhibited the highest antiangiogenic potential in concentration dependent manner, i.e., 51.00 ± 0.58, 55.95 ± 0.24, 64.25 ± 0.24, 70.30 ± 0.89, and 78.05 ± 0.48% at concentrations of 62.5, 125, 250. 500, and 1,000 μg/mL with IC_50_ value of 65.02 μg/mL. Similarly, Eu-EtAc and Eu-Met also exhibited antiangiogenic activity with IC_50_ value of 157.55 and 191.64 µg/mL. All the test samples showed dose-dependent response. The result shown by Eu-Chf was closer to the positive control result. The test samples showed the following order of activity; Eu-Chf > Eu-EtAc > Eu-Met with IC_50_ values of 65.02, 157.55, 191.64 µg/mL, respectively. A summary of the antiangiogenic potential of Eu-Met extract and fractions (Eu-EtAc and Eu-Chf) of *E. umbellata* is presented in Figure 1.

### 3.2 Molecular docking

The identified 12 phenolic compounds (catechin, catechin hydrate, chlorogenic acid, ellagic acid, epigallocatechin gallate, gallic acid, malic acid, mandelic acid, morin, phloroglucinol, quercetin, and rutin) in the Eu-Met, Eu-EtAc, and Eu-Chf act as ligands, their binding affinities and interacting amino acid residues within the active site of VGEFR-2 (3vhe) are shown in Table 2. Binding affinity threshold was established so that only those ligands/compounds were analyzed/studied further which had binding affinity less than −9.0 kcal/mol. Only five compounds had binding affinities less −9.0 kcal/mol, these are catechin, catechin hydrate, morin, quercetin, and rutin.

**Table 2:** Molecular docking study of identified compounds in the extract/fraction of *E. umbellata*

S. no	Compounds	Binding affinities (kcal/mol)	Interacting amino acid residues
1	Catechin	−9.4	CYS A:919, LEU A:840, ASP A:1046, LEU A:1035, CYS A:1045, PHE A:1047, VAL A:848, LYS A:868
2	Catechin hydrate	−9.3	VAL A:916, LYS A:868, VAL A:848, ALA A:866, LEU A:840, VAL A:899, LEU A:1035, CYS A:1045, CYS A:916
3	Chlorogenic acid	−8.3	ASP A:1046, CYS A:1045, ALA A:866, LEU A:1035, PHE A:1047, VAL A:848, HIS A:1026
4	Ellagic acid	−8.0	ILE A:1044, ASP A:1046, LEU A:889, ILE A:892, ILE A:888, VAL A:899
5	Epigallocatechin gallate	−8.1	LYS A:868, ASP A:814, ILE A:888, ASP A:1046, HIS A:1026
6	Gallic acid	−6.2	CYS A:919, LEU A:1035, VAL A:848, LEU A:840, ALA A:866
7	Malic acid	−4.8	LEU A:1029, ALA A:1073, TRP A:1071, SER A:1086, MET A:1072, SER A:1090
8	Mandelic acid	−6.3	ASP A:1046, ALA A:866, CYS A:1045, LEU A:1035, PHE A:1047, VAL A:848
9	Morin	−9.9	CYS A:919, LYS A:868, VYS A:1045, VAL A:848, ALA A:866, LEU A:840, LEU A:1035, PHE A:1047
10	Phloroglucinol	−5.2	CYS A:919, LEU A:840, VAL A:848, ALA A:866, LEU A:1035
11	Quercetin	−10.2	LYS A:868, CYS A:919, LEU A:840, ALA A:866, CYS A:1045, PHE A:1047, LEU A:1035, VAL A:848
12	Rutin	−9.4	ARG A:1027, ALA A:881, ILE A:1025, APS A:1046, ILE A:888, GLY A:1048

**Figure 2:**
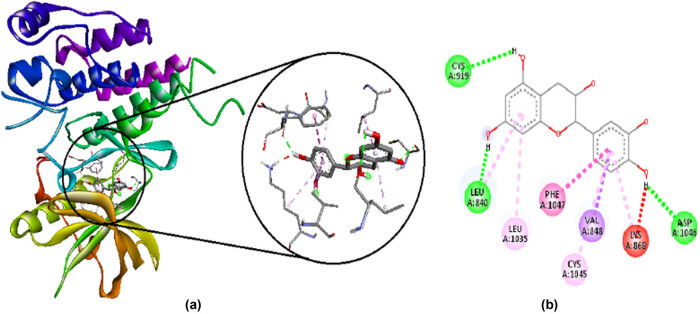
Interaction of catechin with amino acid residues in VEGFR-2 visualized by Discovery studio 2021. (a) 3D interactions and (b) 2D interactions; green shows hydrogen bonds, purple, pink, and light pink are hydrophobic bonds, and red shows unfavorable interaction of acceptor/donor clash.

Catechin has a binding affinity of −9.4 kcal/mol. It makes favorable interactions such as hydrogen bonds with CYS A:919, LEU A:840, and ASP A:1046, and hydrophobic bonds with LEU A:1035, CYS A:1045, and PHE A:1047. It also makes unfavorable interaction of acceptor/donor clash with LYS A:868. Their 2D and 3D interactions are shown in Figure 2.

**Figure 3:**
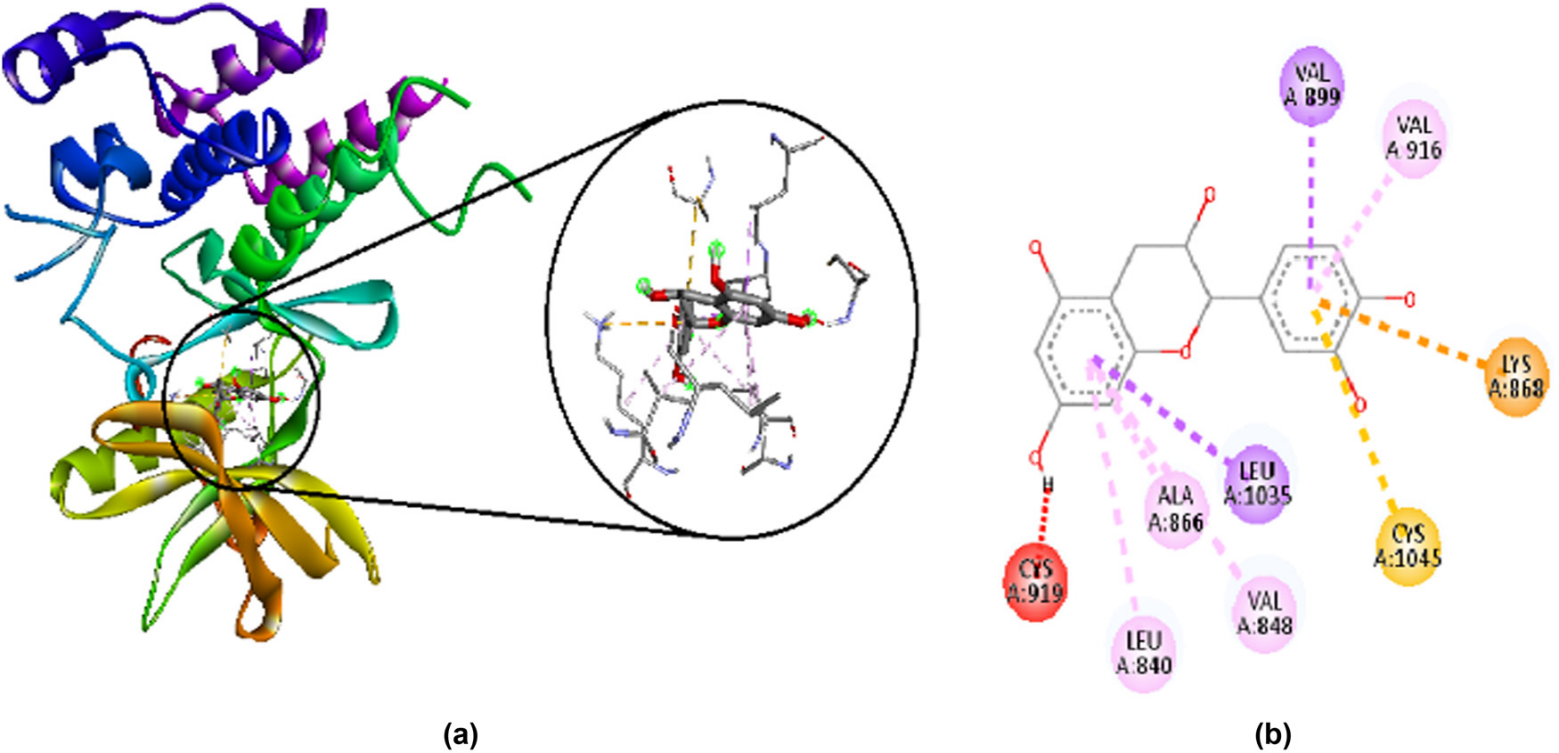
Interaction of Catechin hydrate with amino acid residues in VEGFR-2, visualized by Discovery studio 2021. (a) 3D interactions and (b) 2D interactions; purple and light pink show hydrophobic bonds, orange shows electrostatic bond, yellow shows miscellaneous bonds and red shows the unfavorable interaction of acceptor/donor clash.

Catechin hydrate has a binding affinity of −9.3 kcal/mol. It makes favorable interactions such as electrostatic bonds with LYS A:868, hydrophobic bonds with VAL A:899, LEU A:1035, VAL A:919, LYS A:868, VAL A:848, ALA A:866, and LEU A:840, and miscellaneous bonds with CYS A:1045. It also makes unfavorable interaction of acceptor/donor clash with CYS A:919. Their 2D and 3D interactions along with receptor surfaces are shown in Figure 3.

**Figure 4:**
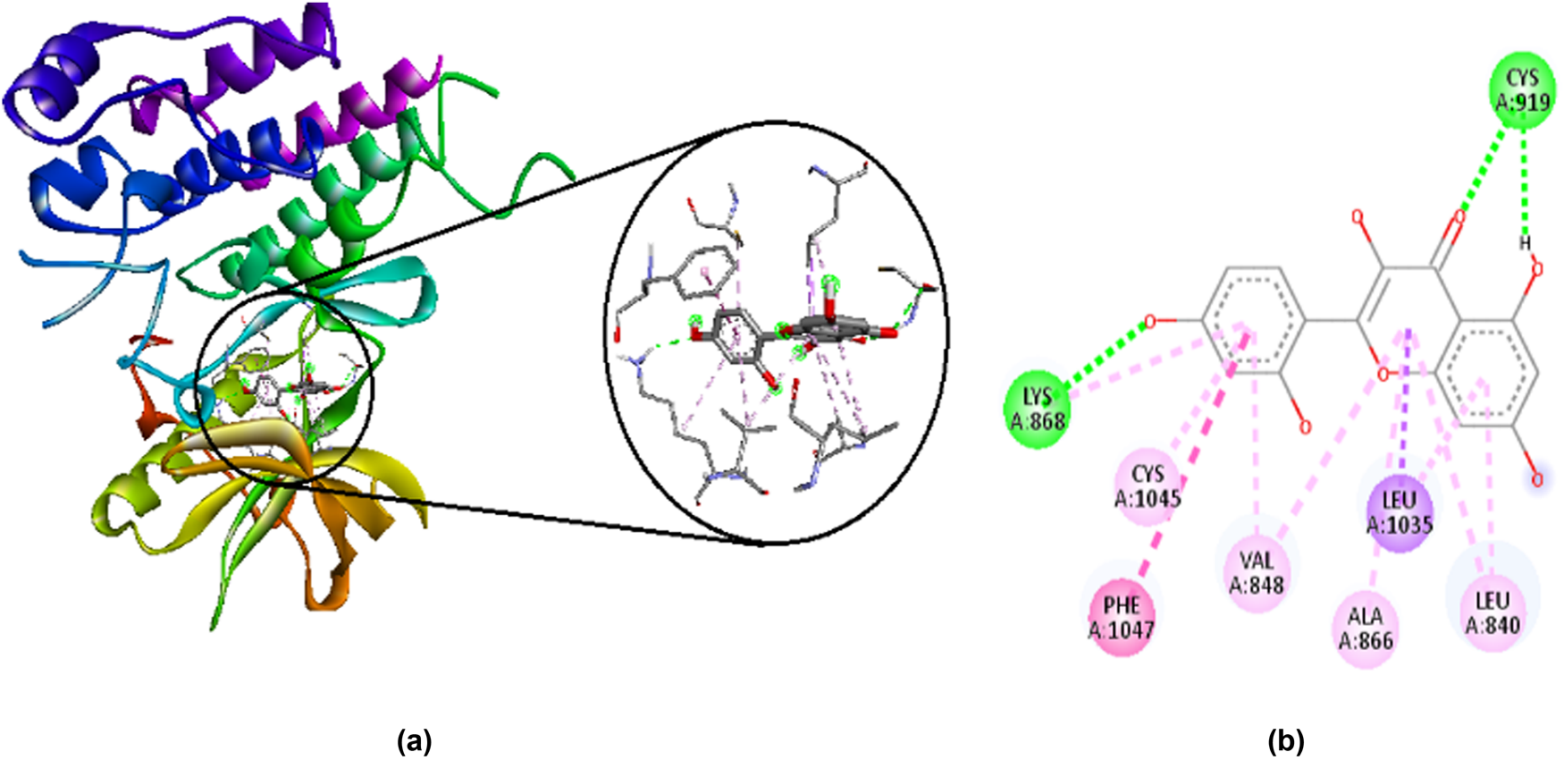
Interaction of morin with amino acid residues in VEGFR-2, visualized by Discovery studio 2021. (a) 3D interactions and (b) 2D interactions; green stand for hydrogen bonds, purple, dark pink, and light pink for hydrophobic bonds, and red for unfavorable interaction of acceptor/donor clash.

Morin has a binding affinity of −9.9 kcal/mol. It makes favorable interactions such as hydrogen bonds with CYS A:919 and LYS A:868, and hydrophobic bonds with LEU A:840, ALA A:866, VAL A:848, CYS A:1045, LYS A:868, LEU A:1035, and PHE A:1047. It also makes unfavorable interaction such as acceptor/donor clash with CYS A:919. Their 2D and 3D interactions along with receptor surfaces are shown in Figure 4.

**Figure 5:**
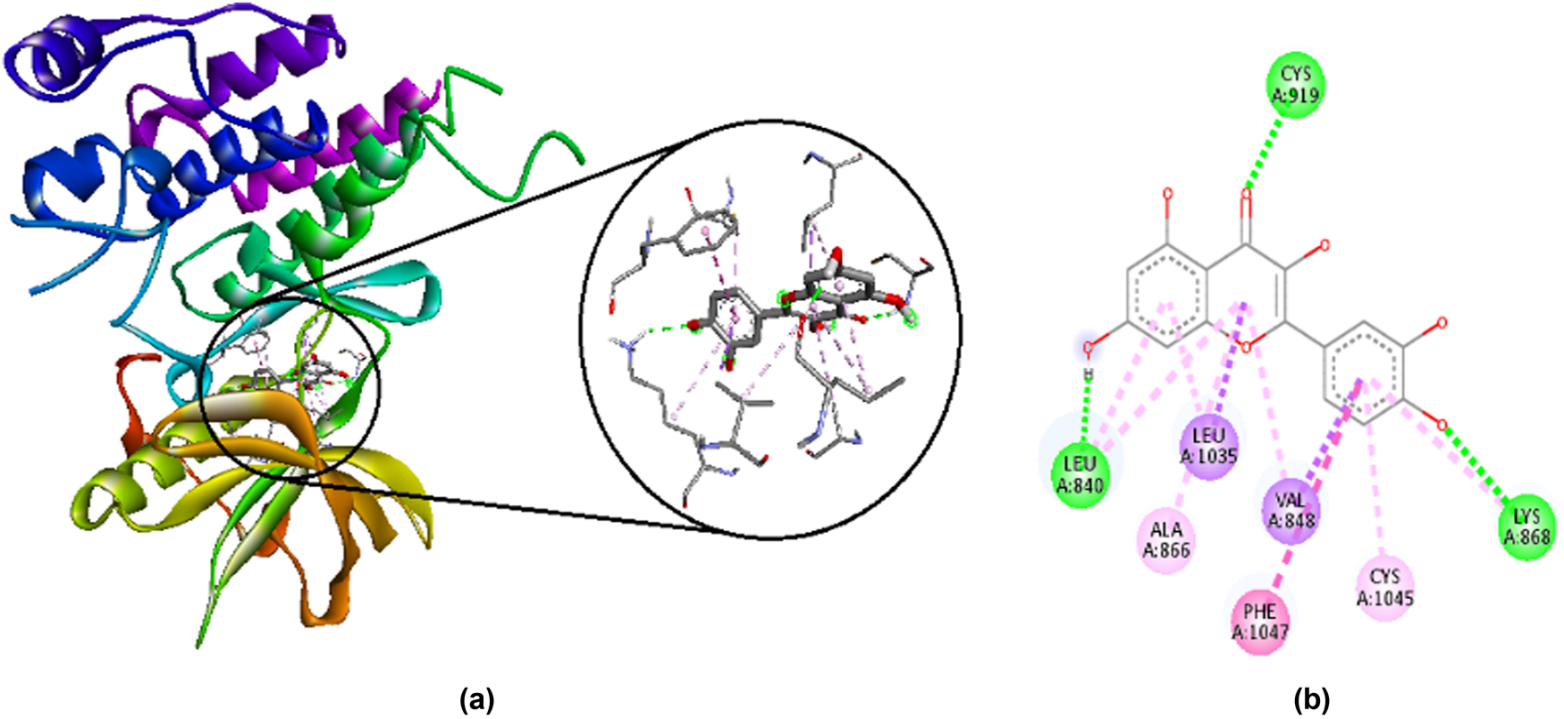
Interaction of quercetin with amino acid residues in VEGFR-2, visualized by Discovery studio 2021. (a) 3D interactions and (b) 2D interactions; green shows hydrogen bonds, purple, dark pink, and light pink show hydrophobic bonds.

Quercetin has a binding affinity of −10.2 kcal/mol. It makes favorable interactions such as hydrogen bonds with CYS A:919, LYS A:868, and LEU A:840, and hydrophobic bonds with LEU A:840, ALA A:866, CYS A:1045, LYS A:868, LEU A:1035, VAL A:848, and PHE A:1047. Their 2D and 3D interactions along with receptor surfaces are shown in Figure 5.

**Figure 6:**
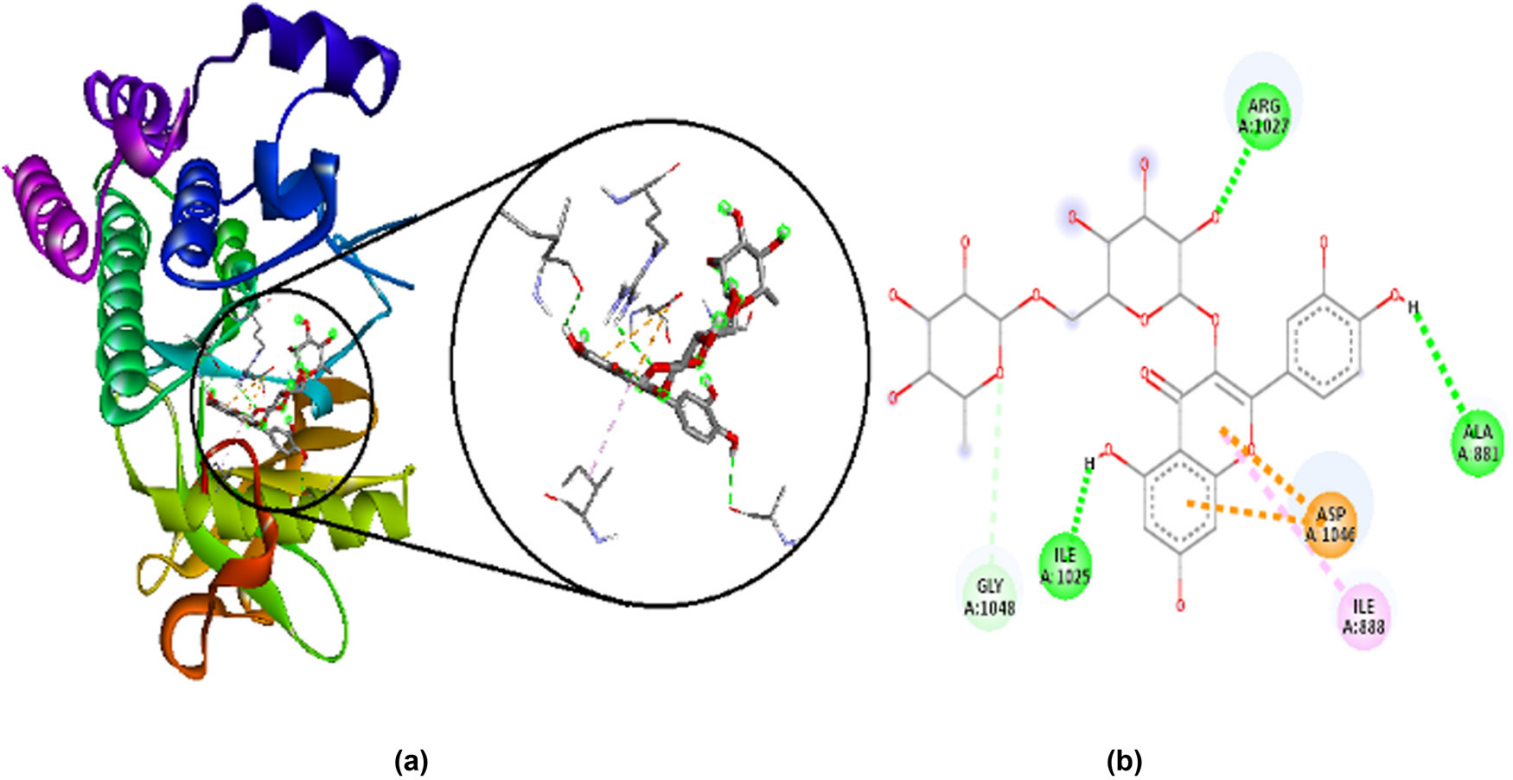
Interaction of rutin with amino acid residues in VEGFR-2, visualized by Discovery studio 2021. (a) 3D interactions and (b) 2D interactions; green and white show hydrogen bonds, while purple and pink show hydrophobic bond, and orange shows electrostatic bond.

Rutin has a binding affinity of −9.4 kcal/mol. It makes favorable interactions such as hydrogen bonds with ARG A:1027, ALA A:881, ILE A:1025, and GLY A:1048, electrostatic bond with ASP A:1046, and hydrophobic bond with ILE A:888. Their 2D and 3D interactions along with receptor surface are shown in Figure 6.

The order of binding affinities of the various ligands/compounds were Quercetin > Morin > Rutin > Catechin > Catechin hydrate > Chlorogenic acid > Epigallocatechin gallate > Ellagic acid > Hexyl Benzene > Mandelic acid > Gallic acid > Phloroglucinol.

## 4 Discussion

Angiogenesis inhibitors block blood vessel growth and starve the tumor cells, by blocking nutrients and oxygen from a tumor [34]. Various plant species have been reported to have antiangiogenic activity [35,36]. The antiangiogenic activity of plants are due to their phytochemicals [37,38]. Various species in Elaeagnus family have antiangiogenic/anticancer activity. Ethyl acetate, ethanol, and water extracts of *Elaeagnus angustifolia* have exhibited strong antiangiogenic activity [39]. *E. angustifolia* extract has also reduced blood vessel development of the CAM assay [39]. A. Nelson has reported *in vitro* and *in vivo* anticancer activity of *Elaeagnus rhamnoides* (L.) [40]. *Elaeagnus multiflora*, *Elaeagnus Angustifolia, Elaeagnus caudata, and Elaeagnus conferta* have all been reported to have anticancer/anti angiogenic activity [41,42,43,44]. *E. umbellata* is a high value medicinal plant with previously reported anti-diabetic, anticholinesterase, and antioxidant activities but no antiangiogenic activity [22]. In this study, we applied CAM assay and molecular docking to study the antiangiogenic potential of *E. umbellata*. Both of these methods provide rapid, economic, and reliable results. In CAM assay, reduction in number of blood vessels were observed by the various extracts in a dose-dependent manner, among them, the extract Eu-Chf showed notable antiangiogenic activity with IC_50_ value of 65.02, which is close to the standard, dexamethasone in Table 1. This antiangiogenic activity is due to the phytochemicals present in the various extracts of *E. umbellata* [20].

To further dwell into the molecular mechanism, molecular docking was conducted by targeting VEGFR-2. VEGFR-2 was selected because VEGF/VEGFR2 interaction activates the phosphorylation cascade that triggers downstream cellular signaling pathways, including the PI3K/AKT and RAF/MAPK pathways which promote angiogenesis and the VEGF/VEGFR2 pathway is the central therapeutic target in antiangiogenic treatment in multiple cancers [4,11]. Furthermore, it has been reported that the inhibition of VEGFR2 not only blocks angiogenesis in tumors but it can also destroy the tumor vessels [45]. Therefore targeting VEGFR-2 might inhibit angiogenesis. The docking results revealed 5 out of 12 compounds, i.e., catechin, catechin hydrate, morin, quercetin, and rutin had binding affinity less than −9.0 kcal/mol (Table 2). These five compounds showed strong interactions with VEGFR-2 present in the various extract of *E. umbellata* [20].

The anticancer and antitumor properties of Quercetin has been reported [46]. Quercetin has been able to inhibit both the translocation and the expression of VEGFR-2 in human umbilical vein endothelial cells [47]. Quercetin has also been found to inhibit angiogenesis in dose dependent manner in human microvascular dermal endothelial cells [48]. Similarly, Quercetin also inhibits the activation of VEGFR‐2, suppressing the Ras downstream cascade of MEK/ERK, MEK/JNK, and PI3‐K/AKT pathways [45]. The anticancer properties of rutin has also been reported [49]. Rutin is demonstrated to inhibit the proliferation of breast, colon, lung, and prostate cancers, and other tumors [50]. However, there is no such study available on direct effect of rutin on VEGFR-2 but it has been reported that rutin exerts its tumor inhibitory effect through the regulation of signaling pathways like Jun N-terminal kinase (JNK), MAPK, p38, PI3K/Akt/mTOR [51]. The epidermal growth factor (EGF) stimulates all the signaling pathways like PI3K/Akt and Ras/Raf, TGF-β2/Smad2/3Akt/PTEN, and mammalian target of rapamycin (mTOR) [50]. Rutin can bind to the EGF receptor protein (EGFR) and obstruct subsequent downstream signaling pathways [50], so rutin might also inhibit VEGFR-2 and its downstream signaling pathways. Green tea catechin has also been reported to have anticancer activity and can interfere with VEGFR-2 [52,53]. Morin possesses potent anticarcinogenic and anticancer activities with minimal toxicity against normal cells and a variety of molecular targets and signaling pathways such as apoptosis, cell cycle, reactive oxygen species, etc., as well as signal transducer and activator of transcription 3, nuclear factor kappa B, PI3K/Akt, MAPK, and Hippo pathways are involved in the anticancer effects of morin [54]. Morin is a also good therapeutic candidate for the treatment of HER2-overexpressing breast cancer as it induces cell death by inhibiting the HER2/EGFR signaling pathway [55]. The morin/CD inclusion complexes have high potential for angiogenesis-dependent disease treatment [56].

Literature reports that these substances – catechin, catechin hydrate, morin, quercetin, and rutin – have anticancer properties. The extract/fractions of *E. umbellata* have demonstrated antiangiogenic activity, which could be attributed to the presence of these phytochemicals. Our study has various limitations that should be acknowledged. In this study, we only used a simple antiangiogenic model via CAM assay and molecular docking to demonstrate the interactions of these phytochemicals with the active site of VGEFR-2. To confirm *E. umbellata’s* antiangiogenic efficacy, more advanced *in vivo* and *in vitro* experimental models should be adopted. Second, the interaction between VEGFR-2 and specific phytochemicals is explored *in silico* using molecular docking; therefore, the specific phytochemicals must be extracted and purified from *E. umbellata*, and their effect on the VEGFR-2 and cell signaling pathways must be assessed.

## 5 Conclusion

Based on our results, extracts of *E. umbellata* were able to inhibit the CAM assays’ blood vessel development and the identified compounds catechin, catechin hydrate, morin, quercetin, and rutin were able to bind strongly to VEGFR-2 in molecular docking study which needs to be explored further. This study revealed that the phytochemicals of *E. umbellata* have the potential to bind to and inhibit VEGFR-2. It has been demonstrated that five compounds (catechin, catechin hydrate, morin, quercetin, and rutin) have strong interactions with VEGFR-2 having high binding energies. A significant number of hydrogen bonds and hydrophobic interactions were formed by each of these compounds with VEGFR-2. Thus, it can be concluded that these identified compounds serve as good building blocks for the development of a specific VEGFR-2 inhibitor. These VEGFR-2-targeting drugs will influence the inhibition of angiogenesis in many cancer forms. Further studies employing *in vivo* models would be required to confirm their therapeutic utility in the treatment of cancer.

## Abbreviations

AKT: protein kinase B
CAM: chorioallantoic membrane
EGF: epidermal growth factor
EGFR: epidermal growth factor receptor
JNK: Jun N-terminal kinase
MAPK: mitogen-activated protein kinases
mTOR: mammalian target of rapamycin
PDB: protein databank
PDBQT: Protein Data Bank, Partial Charge (Q), & Atom Type (T)
PI3K: phosphatidylinositol-3 kinase
RAF: rapidly accelerated fibrosarcoma
RAS: rat sarcoma
SMAD2: Mothers against decapentaplegic homolog 2
TGF: transforming growth factor
VEGF: vascular endothelial growth factor
VEGFR-2: vascular endothelial growth factor receptor 2
